# Factors, mechanisms and improvement methods of muscle strength loss

**DOI:** 10.3389/fcell.2024.1509519

**Published:** 2024-12-04

**Authors:** Kaiyong Wang, Xuyu Wang, Yanqiu Wang

**Affiliations:** ^1^ Department of Physical Education, Guangdong University of Finance and Economics, Guangzhou, Guangdong, China; ^2^ Master program under the Graduate School of Education, Graduate University of Mongolia, Ulaanbaatar, Mongolia; ^3^ School of Physical Education, Central China Normal University, Wuhan, Hubei, China

**Keywords:** muscle strength loss, factors, muscle disorders, muscle strength assessment, exercise

## Abstract

Muscle strength is a crucial aspect of muscle function, essential for maintaining normal physical activity and quality of life. The global aging population coupled with the increasing prevalence of muscle disorders and strength loss, poses a remarkable public health challenge. Understanding the mechanisms behind muscle strength decline is vital for improving public health outcomes. This review discusses recent research advancements on muscle strength loss from various perspectives, including factors contributing to muscle strength decline, the signaling pathways involved in the deterioration of muscle function, and the methods for assessing muscle strength. The final section explores the influence of exercise stimulation and nutrition on muscle strength.

## 1 Introduction

The decline in organ function is an inevitable part of aging. The global population aged 65 and older is projected to rise from 9% at present to 16% by 2050 (Gu et al., 2020; Chen et al., 2024), which poses considerable public health challenges. This demographic shift, along with the rising prevalence of age-related diseases, is not only a serious public health concern but also a substantial economic burden. Skeletal muscle, which accounts for approximately 40% of body weight, plays a critical role in physical activity and overall quality of life, with muscle strength being a key component of muscle function. Muscle strength naturally declines in the aging process, and this decline is also associated with various muscle disorders. Moreover, muscle strength is linked to all-cause mortality and an increased risk of cardiometabolic diseases. To some extent, muscle strength serves as an indicator of body composition and general health status (Carbone et al., 2020).

Multiple factors, including sarcopenia and disuse, have been clinically investigated as contributing factors to the development of muscle strength loss. The molecular mechanisms and signaling pathways involved in muscle hypertrophy and atrophy, studied using diverse animal models, are a key focus of basic research. In addition to protein turnover and muscle fiber morphology, the exploration of muscle stem cells and their niche has provided shed insights into the physiology and pathology of skeletal muscle, offering potential treatment strategies for muscle disorders. In practical terms, exercise training and nutritional interventions have shown positive effects in improving muscle function including, muscle strength.

This review discusses muscle strength loss, its potential contributing factors, the signaling pathways involved, and clinical assessment methods for muscle strength loss. Additionally, studies on the effects of exercise training and nutritional interventions in healthy populations and patients on muscle strength were highlighted to emphasize their importance in clinical practice.

## 2 Factors associated with muscle strength loss

Body movements depend on the musculoskeletal system, which comprises skeletal bones, connective tissue, and skeletal muscles. Skeletal muscles are attached to bones via tendinous tissue and generate body movements through contraction (Kerkman et al., 2018). In addition to enabling physical activity, skeletal muscles also play a role in nutrient storage and energy use. The dynamic nature of skeletal muscles is reflected in their diverse metabolic patterns, which vary between periods of rest and exercise (Smith et al., 2023).

Healthy skeletal muscle with effective force-generating capacity is necessary for daily activities and athletic performance. A comprehensive understanding of the mechanisms that maintain excellent muscle quality and attenuate the decline of muscle function under pathological conditions is crucial for promoting health span. Muscle performance is determined by muscle quantity and muscle quality. Muscle quality, which refers to the functional capacity of muscle, is assessed by the amount of strength generated per unit of muscle mass (Naimo et al., 2021). While muscle mass is often regarded as a key factor in force generation, the relationship between muscle mass and muscle performance can be complicated. Clinical evidence, particularly in older adults, has shown that some studies found no significant association between muscle mass loss and declines in physical performance (Riviati and Indra, 2023). Recent clinical observations are challenging the view that muscle mass is the only criterion for assessing muscle health. If reduced muscle mass does not fully explain the decline in physical performance, then other factors must contribute to the changes associated with disease progression and aging. Muscle quality encompasses various properties of muscle, including force production, composition, and metabolism. Compared with the concept of muscle quantity, muscle quality focuses on skeletal muscle as a complex, dynamic entity with whose metabolism is regulated by physical activity, as well as by internal inflammatory processes and the cellular microenvironment (Koo, 2022).

**FIGURE 1:**
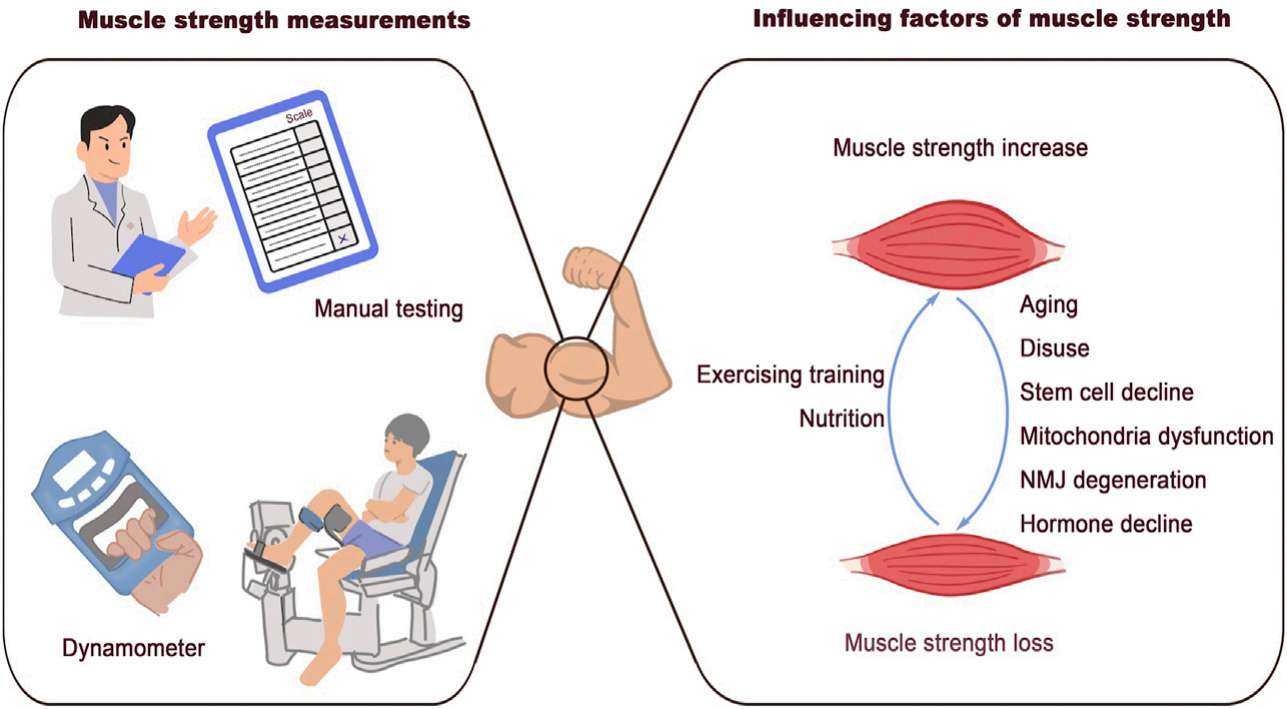
Associated factors of muscle strength loss. Clinical methods for muscle strength measurement: manual testing and dynamometer. The discussed factors that are associated with muscle strength loss: aging, disuse, stem cells decline, mitochondrial dysfunction, NMJ degeneration, and hormone decline.

Muscle strength is defined as the maximum voluntary force that muscles can exert on the environment under specific test conditions. The diverse properties involved in generating muscle strength indicate that numerous factors contribute to its loss. These factors are summarized in Figure 1.

### 2.1 Aging and muscle strength loss

Among all the factors contributing to muscle strength loss, aging is the most unavoidable. Particularly, those aged 75 years or older experience a muscle loss rate of 0.64%–0.7% per year (Miljkovic et al., 2015). The term sarcopenia was initially introduced to describe the reduction of lean body mass associated with aging (Rosenberg, 1997). Over time, the definition of sarcopenia has evolved to encompass not only muscle loss but also muscle weakness and impaired performance (Miljkovic et al., 2015). Muscle mass beings to decline in middle age due to muscle fiber atrophy and loss. An imbalance between muscle protein synthesis and breakdown is known to lead to muscle fiber atrophy (Wilkinson et al., 2018). Studies comparing muscle fiber size in younger and older populations indicate that the reduction in muscle fiber size in older individuals may be specific to muscle fiber type. Compared to young adults, type II muscle fibers in older adults are 10%–40% smaller, while the size of type I muscle fiber shows no substantial difference between the two age groups (Verdijk et al., 2007; Snijders et al., 2009; Deschenes, 2004; Nilwik et al., 2013). Muscle fibers are generally classified as type I (slow-switch fibers) and type II (fast-switch fibers). This classification and its implications for muscle function and performance has been extensively described elsewhere (Scott et al., 2001). The different types of muscle fibers, each with unique properties and metabolic profiles, are essential for various types of physical activity, including resistance and endurance exercises (Qaisar et al., 2016). Type II fibers are mainly responsible for high-intensity activities, while type I fibers are required for endurance activities. The decline in type II fiber size with aging partly explains the loss of muscle strength observed in older adults. However, the reduction in muscle mass is not the only change that occurs with aging. The capability of individual muscle fibers to generate force also diminishes in the elderly. The concept of specific force refers to isometric muscle strength normalized to the cross-sectional area of muscle fibers. A decrease in specific force in older adults reveals a reduction in muscle quality, rather than only a loss of muscle mass (Young et al., 1985). *In situ* examination of force generation of skeletal muscle of young and old rats indicated that the decreased muscle quality and contractile force may be attributed to the impaired sarcoplasmic reticulum Ca^2+^ release in the aged muscles (Russ et al., 2011). In addition to the reduction in muscle fiber size, changes in the composition and structure of muscle fibers also contribute to the decline in muscle quality as we age. Myosin and actin are the major proteins in the myofilament, and their coordination is essential for muscle contraction and efficient force generation within a single fiber. Studies examining biopsy samples from young, elderly, and immobilized elderly individuals have shown that the specific force of single muscle fibers notably decreases in older adults and further declines in immobilized elderly individuals. Additionally, the concentration of myosin in muscle fibers was found to be consistently altered in parallel with the decrease in specific force (D'Antona et al., 2003). The regulation of myofiber proteins in aging, as well as in other pathological conditions, will be extensively discussed in the subsequent section on mechanisms.

### 2.2 Muscle stem cells and muscle degeneration

Muscle satellite cells, also known as muscle stem cells, are crucial for maintaining muscle homeostasis and facilitating regeneration. The biological functions of satellite cells have been intensively studied, particularly in the context of muscle regeneration and aging. Animal models, such as zebrafish, mice, and rats, have been instrumental in advancing our understanding of the role of muscle stem cells during aging at the molecular and cellular levels (Porpiglia et al., 2022). Research has shown that the number of satellite cells decreases as muscle ages, and this decline is closely associated with muscle aging (Munoz-Canoves et al., 2020). The regulation of the satellite pool during aging involves complex changes in the cell cycle and cell niche (Chen et al., 2020). In addition to the reduction in the number of satellite cells, their function and metabolic state also undergo irreversible changes with aging (Memczak and Belmonte, 2023). Studies in adult sedentary mice with satellite cell depletion have shown that while regeneration capacity decreases and fibrosis increases, no accelerated progress of sarcopenia or considerable alteration in fiber type composition was found (Fry et al., 2015). This result indicates that, in sedentary mice, the lifelong depletion of muscle stem cells does not affect the maintenance of muscle fiber size. Aged satellite cells have been known to contribute to the decreased regeneration capacity in older individuals. However, the exact mechanisms by which aged satellite cells influence muscle quality and lead to decreased muscle strength need additional investigation.

### 2.3 Intermuscular fat influences muscle quality

Muscle strength may not depend solely on skeletal muscle tissue itself. Fat infiltration has gained considerable attention from researchers and clinicians. A longitudinal study demonstrated an increase in intermuscular fat in older adults, along with a decrease in muscle quality (Delmonico et al., 2009). Intermuscular adipose tissue refers to fat infiltration within the fascial envelope of the muscle. This fat infiltration is regarded as an influential factor of muscle metabolism in the elderly, which may negatively affect the force production of muscles (Marcus et al., 2010). A study involving 54 healthy male individuals aged 20–70 in Europe found that, with muscle atrophy during aging, some muscle tissue was replaced by adipose tissue, and the fat content in the remaining muscle increased (Engelke et al., 2022). The accumulated fat does more than simply replace muscle tissue. Substrates secreted by adipose cells contribute to the creation of an inflammatory immune microenvironment within the muscle tissue, which, in turn, promotes the breakdown of muscle function, including a reduction in force production (Li C. W. et al., 2022).

### 2.4 Nerve and neuromuscular junction in muscle aging

Skeletal muscle, which is responsible for body movements, initiates force generation through regulation by the nervous system. The neuromuscular junction (NMJ), a synaptic structure, is the key element bridging the motor nerve and muscle fibers. This structure comprises the motor nerve terminal in the pre-synaptic region, the synaptic basal lamina in the intrasynaptic space, and the muscle fiber and membrane in the post-synaptic area (Gonzalez-Freire et al., 2014). With aging, skeletal muscle experiences irreversible neuron loss (Glass and Roubenoff, 2010). Age-associated pathological changes in the peripheral nerve and NMJ are considered key factors in muscle degeneration associated with sarcopenia (Deschenes et al., 2010). Denervation of skeletal muscle and functional deterioration of the NMJ during aging not only lead to muscle mass loss but also contribute to the reduction of muscle quality (Iyer et al., 2021).

Overall, in addition to the reduction in muscle mass in aging, the aging of myofibers and the alterations of the microenvironment in the skeletal muscle tissues also contribute to weak muscle performance and strength generation in the elderly.

The conventional definition of sarcopenia primarily refers to the loss of muscle mass and strength in the elderly. However, sarcopenia in younger populations is drawing increasing global attention. Although diagnostic criteria for sarcopenia in adolescents and young individuals are not yet fully established, a lack of comprehensive clinical data and the use of varied assessment tools led to increasing concern and emerging studies on sarcopenia in younger individuals (Jung et al., 2023; Hwang, 2023).

### 2.5 Muscle injury and repair affect muscle quality

Skeletal muscle injury resulting from daily physical activities, exercise training, or trauma is typically followed by an immediate loss of muscle strength (Warren et al., 2017). Based on their causative factors, muscle injury can be divided into intrinsic and external injuries. Externally induced muscle injuries may result in contusions or lacerations, while internally induced injuries often lead to muscle strains. Contusions and strains account for more than 90% of sports-induced muscle injuries, whereas lacerations are less common (Järvinen et al., 2005). Regarding the severity of muscle injury, grade I represents a mild tearing of a few muscle fibers with minimal loss of function. A grade II injury involves a more substantial tissue damage and a greater loss of function compared to grade I. A grade III injury is the most severe, characterized by a complete tear of the skeletal muscle and total loss of function. Muscle rupture is classified as a grade IV injury (Hamilton et al., 2015). Similar to most tissue injuries, skeletal muscle injury progresses through several phases: inflammation, repair, regeneration, and remodeling (Baoge et al., 2012). During the regeneration phase, necrotic tissues is cleared through phagocytosis, myofibers regenerate, and revascularization occurs. However, along with this regeneration, connective tissue scarring may also form. In the remodeling phase, newly regenerated myofibers mature, and the connective tissue reorganizes as the muscle function is restored (Jarvinen et al., 2007). In the initial stage of muscle injury, muscle fibers rupture and necrose, and the hematoma forms, which directly disrupt the structure of the muscle’s basic unit for force generation. Theoretically, once muscle fibers regenerate and the skeletal muscle undergoes repair, muscle strength should recover. However, the tissue cannot always fully recover its original morphology after an injury due to the reorganization of regenerated myofibers and the formation of scars. Fibroblasts, immune cells such as macrophages, and the factors released by them have been shown to play a key role in the pathological deposition of extracellular matrix and the formation of fibrotic tissue (Gardner et al., 2020).

### 2.6 Muscle disuse and muscle strength loss

Active mobilization immediately after acute muscle injury may result in large permanent scar tissues at the trauma site. By contrast, appropriate mobilization after initial immobilization is necessary for rehabilitation. Notably, the rehabilitation activity should be initiated in accordance with the clinicians after muscle injury for muscle function recovery (Järvinen et al., 2013). In chronic muscle diseases, such as Duchenne muscular dystrophy and other dystrophies, the lack of regeneration efficiency due to dysregulation of satellite cells in the skeletal muscle allows for the replacement of muscle tissue by adipose and fibrotic tissues (Mann et al., 2011). In addition to gradual rehabilitation activity, targeting the cytokines from inflammatory cells may also help intervene the fibrosis after acute muscle injury or chronic muscle disease (Tidball and Wehling-Henricks, 2015; Laumonier and Menetrey, 2016; Garg et al., 2015).

Patients with muscle disuse face accelerated muscle mass degradation, bringing the decline in muscle strength from 0.3% to 4.2% per day (Wall and van Loon, 2013). The impact of disuse on muscle strength can be rapid and profound. According to a study involving 24 healthy young male individuals, muscle cross-section area and muscle strength markedly decreased after only 5 days of disuse of single-leg, while the muscle mass showed a remarkable decrease after 14 days of disuse (Wall et al., 2014). Numerous clinical settings, including bed rest, joint immobilization, tissue unloading, and spinal cord injury, could lead to the disuse of skeletal muscles (Bodine, 2013). Disuse of muscles has also been associated with muscle loss and impaired force production (Rudrappa et al., 2016; Mirzoev, 2020; Nunes et al., 2022).

### 2.7 Hormone and muscle quality

Hormones, secreted by multiple tissues, regulate growth and development by binding to their receptors throughout the body. Growth hormone (GH) and insulin-like growth factor (IGF) are key anabolic regulators of tissue growth during development (Ranke and Wit, 2018). The decline in hormone levels with aging is another crucial factor of influencing muscle quality. In women, menopause leads to a reduction in estrogen and progesterone production, which affects various organs (Gatenby and Simpson, 2024). In men, andropause refers to a gradual process of hypogonadism, characterized by a decline in testosterone levels (Kadiwala and Dhadwad, 2024). GH exerts an anabolic effect by regulating the expression of its target genes. IGF-1 is one of the most important mediators of GH in promoting the growth of multiple tissues, including skeletal muscle (Chia, 2014). By targeting the liver, the main producer of IGF-1, GH regulates IGF-1 production (Yakar et al., 2002). Adults with GH deficiency (GHD) show decreased muscle mass (Chikani and Ho, 2014). GH replacement can improve reduced lean mass by stimulating protein synthesis and inhibiting protein catabolism (Shi et al., 2003). GH also regulates muscle homeostasis independent of IGF-1. In a study utilizing mice with knockout of Nfatc2, a regulator of muscle cell fusion, GH was found to promote skeletal muscle cell fusion without the upregulation of IGF-1 (Sotiropoulos et al., 2006). Testosterone is the principal androgen produced by Leydig cells in men. This hormone exerts anabolic effects and promotes muscle fiber hypertrophy in skeletal muscle, mainly through interactions with androgen receptors (ARs) (Sinha-Hikim et al., 2002; Vingren et al., 2010). Clinical observations have reported that the decline in testosterone, resulting from aging or androgen deprivation therapy (ADT), generally leads to a notable decrease in muscle mass and strength (Shigehara et al., 2022). Strategies to counteract decreased testosterone levels include testosterone supplement and selective androgen receptor modulators (SARMs), which exert their effects by acting on the ligand and the receptors, respectively (Bhasin et al., 2023). Some clinical trials have reported that testosterone supplementation is sufficient to increase lean body mass and grip strength in older men (Mauvais-Jarvis and Lindsey, 2024; Page et al., 2005; Storer et al., 2017). However, the extent of improvement due to testosterone supplementation varies depending on the specific circumstances of the subjects and the treatment protocols used (Sinha-Hikim et al., 2002; Storer et al., 2017; Snyder et al., 2016; Storer et al., 2016). Primarily produced and secreted by the ovaries, estrogen is responsible for the development and maintenance of reproductive and non-reproductive organs (Coyle-Asbil et al., 2023). Postmenopausal women have been shown to have lower muscle mass and cross-sectional areas compared to premenopausal women (Critchlow et al., 2023). Estrogen deficiency leads to a decline in muscle mass and strength (Yoh et al., 2023). While decreased estrogen levels are associated with reduced muscle quality, clinical observations have found it challenging to improve muscle strength and performance in postmenopausal women through estrogen hormone therapy (Javed et al., 2019; Xu et al., 2020; Rolland et al., 2023). In addition to GH and sexual hormones, several other hormones, including thyroid hormone and cortisol, plays a crucial role in the regulation of skeletal muscle (Kraemer et al., 2020; Nappi et al., 2024). Additional clinical trials are required to examine the effectiveness and safety of hormone replacement therapy and to improve muscle quality and performance in the aging population.

In addition to the aforementioned conditions, pathological conditions in tissues outside of skeletal muscle can also influence muscle quantity and quality. Metabolic diseases, such as diabetes and obesity, have a global impact on muscle tissues. Accelerated loss of skeletal muscle mass and strength has been observed in subjects with type 2 diabetes (Park et al., 2009; Park et al., 2007). While obese individuals tend to have higher absolute muscle strength, their strength is relatively lower when normalized to body mass compared to their non-obese counterparts (Tomlinson et al., 2016; LaRoche et al., 2011). The relatively lower strength and physical performance in obese individuals, especially for the old subjects, is proposed to be a result of systemic inflammation (Tomlinson et al., 2014). Rheumatoid arthritis (RA), another disease with chronic inflammation, may lead to loss of muscle mass and strength (de Oliveira Nunes Teixeira et al., 2013). The development process of RA is accompanied by joint destruction, impaired physical activity, musculoskeletal dysfunction, and even cardiovascular diseases. All these integrative factors influence the homeostasis of skeletal muscles and eventually reduce muscle mass and function, including strength (Yun et al., 2021). The prevalence of sarcopenia in individuals with RA is more than 25%, and rheumatoid sarcopenia is associated with an increased risk of falls. Chronic inflammation mediated by inflammatory factors such as TNF and IL-6 resulted in muscle protein breakdown, and other abnormalities are related to rheumatoid sarcopenia (Bennett et al., 2023).

## 3 Mechanisms of muscle strength loss

### 3.1 Structure of skeletal muscle

**FIGURE 2:**
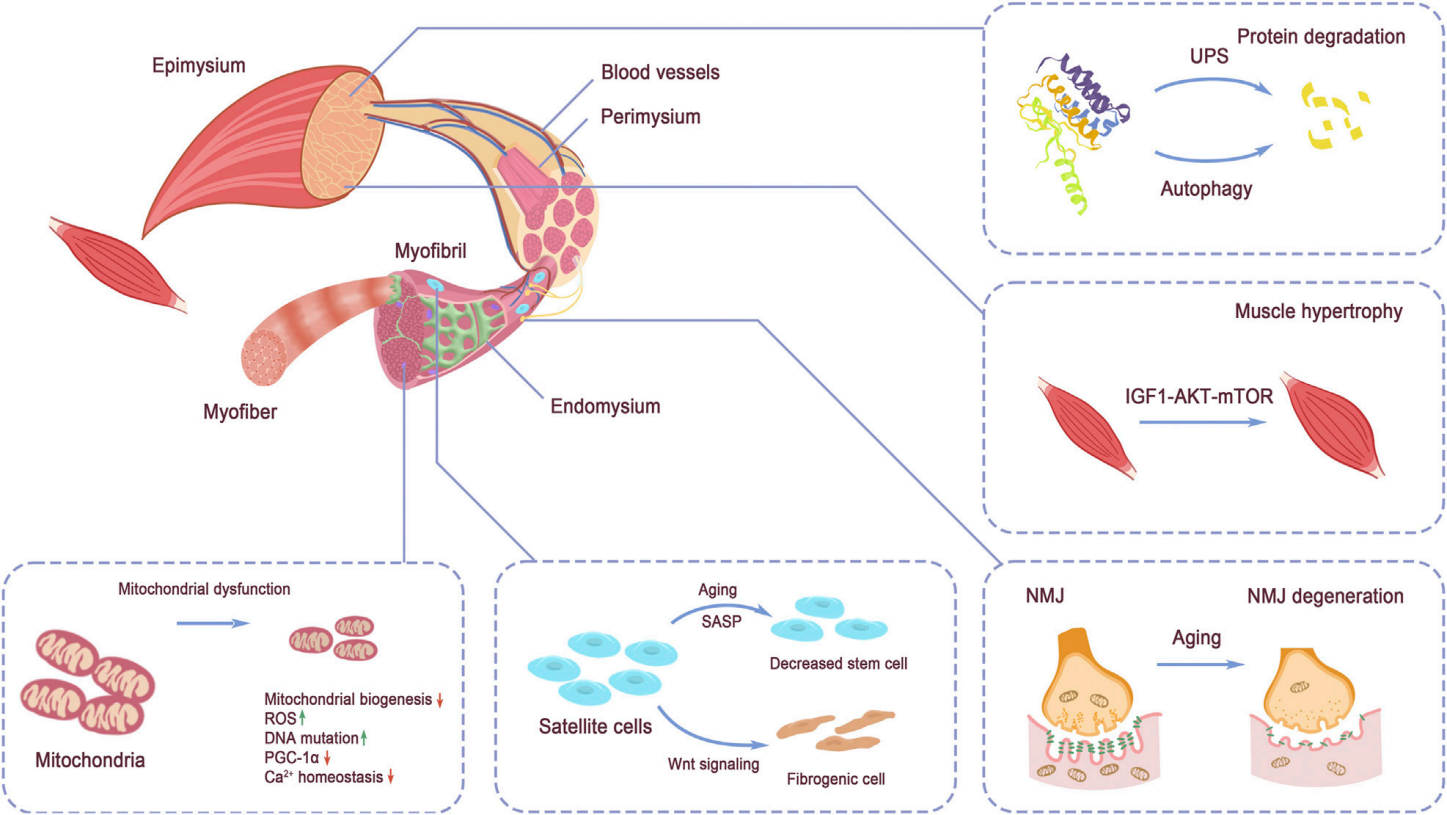
Structure of skeletal muscle. The tissue structure of skeletal muscle and the discussed signaling pathways regulating muscle strength: protein degradation by UPS and autophagy, IGF-1 signaling, NMJ structure, mitochondrial homeostasis, and muscle stem cell pool maintenance. Protein degradation via UPS and autophagy and protein synthesis regulated by IGF-1 signaling balance the protein turnover in muscle. Mitochondria dysfunction directly impairs energy supply in muscle force production. NMJ function decline hinders the exertion of muscle contraction. Stem cell pool decreases and quality alteration disadvantage muscle maintenance and injury repair.

Controlled by the nervous system, over 500 skeletal muscles support body movements by cooperating with bones and tendons (Tieland et al., 2018). The quality of muscle fibers directly influences the performance of skeletal muscles. Within each muscle fiber, the sarcomere serves as the functional unit, enabling muscle contraction and relaxation. Structurally, skeletal muscles are highly organized, with a clear arrangement of tissues. Muscle tissue comprises bundles of fascicles, and within each fascicle are individual myofibers (Mukund and Subramaniam, 2020). The maturation of myotubes, derived from the differentiation and fusion of myoblasts, leads to the formation of myofibers (Trovato et al., 2016). Intramuscular connective tissues, rich in abundant extracellular components, including endomysium, perimysium, and epimysium, are responsible for the segregation of structural units and contain non-muscle tissues, with the endomysium lying between myofibrils. The perimysium surrounds the myofiber, and the epimysium envelopes the entire muscle, defining its overall volume (Purslow, 2020). The basic structure of muscle is illustrated in Figure 2.

#### 3.1.1 Mitochondria in skeletal muscles

In addition to the basic fibers and connective tissues, several other cell types and structures are essential for supporting the motor function and energy production of skeletal muscles. Two subpopulations of mitochondria exist in skeletal muscle. The mitochondria, located beneath the sarcolemmal membrane, are primarily responsible for producing energy required for membrane transport and gene transcription. The other subpopulation, intermyofibrillar mitochondria, is found between the myofibrils and provides ATP for muscle contraction (Hood et al., 2019).

#### 3.1.2 Muscle stem cells in skeletal muscles

Muscle stem cells, also known as satellite cells, are located on the surface of myofibers, between the myofiber plasmalemma and the basal lamina (Danoviz and Yablonka-Reuveni, 2012). The balance between quiescence and activation of satellite cells is crucial for the regeneration capacity of skeletal muscle. Advances in single-cell resolution multi-omics collection technologies have accelerated the investigation of tissue stem populations and their niches. Recently, the muscle stem cell niche, especially the immune microenvironment, has gained increasing attention in multiple animal models, revealing the complexity of the regulatory mechanisms that govern the maintenance and function of satellite cells (Shang et al., 2020; Ratnayake et al., 2021).

#### 3.1.3 Blood vessels and nerves in skeletal muscles

Primary arteries run along the muscles, with arterioles branching off to penetrate the perimysium, eventually branching into capillaries. These blood vessels are responsible for supplying blood flow and facilitating material exchange within the skeletal muscle cells (Pepe and Albrecht, 2023). Additionally, the innervation of skeletal muscles by motor nerves, through the intermediate NMJs, plays a crucial role in regulating muscle function and coordinating muscle contractions (Rudolf et al., 2024).

### 3.2 Mechanisms and signaling pathways in muscle strength loss

Skeletal muscles exhibit remarkable plasticity, enabling the musculoskeletal system to adapt to the changing physical demands of the body. This adaptability relies on the rapid response of skeletal muscles to physical stimuli, which is facilitated by sensitive cellular signaling and transduction pathways.

In the elderly, an imbalance in protein turnover occurs, where protein breakdown gradually exceeds protein synthesis. This imbalance undermines the physiological basis of muscle mass and function, contributing to the age-related decline in muscle health and strength (Wu et al., 2023).

#### 3.2.1 IGF1–AKT–mTOR

Insulin/insulin-like growth factor 1 (IGF1)–AKT–mTOR is the key signaling pathway that regulates protein synthesis and degradation, energy production, and muscle growth. The isoform IGF-1a promotes muscle hypertrophy in young and old mice and protects against the age-related loss of muscle mass and strength (Xu et al., 2020). As one of the downstream elements of insulin/IGF-1 signaling, the kinase mTOR also integrates other stimuli, including cytokines and energy levels (Saxton and Sabatini, 2017). The two key complexes involving mTOR, containing Raptor and Rictor, are responsible for regulating muscle growth and homeostasis. A deficiency in mTORC1 leads to muscle dystrophy and impaired oxidative capacity due to the failure to activate downstream signaling (Bentzinger et al., 2008), while mTORC2 plays a role in the metabolic regulation of skeletal muscle (Yasuda et al., 2023).

#### 3.2.2 Protein degradation in skeletal muscles

The ubiquitin–proteasome system (UPS) and autophagy are the two main approaches responsible for protein degradation (Li Y. et al., 2022). In the UPS pathway, degradation involves ubiquitin and proteasomes. In skeletal muscles, MAFbx and MuRF1 are regarded as the main ubiquitin ligases; however, deficiency in MuRF1 does not fully protect muscles from atrophy induced by denervation or low gravity (Bodine et al., 2001; Cadena et al., 2019). Dysregulation of autophagy impairs the metabolism and homeostasis of multiple tissues, including skeletal muscle (Kitada and Koya, 2021; Lim et al., 2014). Many additional signaling pathways and proteins involved in muscle protein degradation remain undiscovered. In addition to internal regulation abnormalities, muscles have been shown to become resistant to external anabolic stimuli, including resistance exercise and dietary nutrition, as a result of aging (Kim et al., 2020; Boirie, 2009).

#### 3.2.3 Muscle stem cells and muscle function decline

Muscle stem cells, which are central to muscle regeneration, play a critical role in muscle function, particularly during injury repair. In addition to the decline in regenerative capacity, the reduced number of satellite cells with associated with aging is believed to contribute to the degeneration of muscle function in the elderly (Brack et al., 2005; Shefer et al., 2006). When stimulated by injury, satellite cells transform from a quiescent state to an activation state, where they proliferate and differentiate into myoblasts to facilitate muscle repair (McKinnell et al., 2005). The transcription factor Pax7 is a crucial marker and regulator of muscle stem cells, expressed in undifferentiated stem cells and declining as the cells differentiate into progenitors (Rahman et al., 2023). Wnt signaling plays a role in the conversion of myogenic cells to fibrogenic cells during aging (Biressi et al., 2014; Brack et al., 2007). Additionally. Inhibition of JAK–STAT signaling has been demonstrated to promote the function of satellite cells. For example, the injection of JAK–STAT inhibitors can promote muscle regeneration (Price et al., 2014). P38 MAPK signaling has also been found to play a role in regulating satellite cells during aging (Bernet et al., 2014). Furthermore, the functional decline of muscle stem cells can be attributed to the accumulation of aberrant proteins, oxidative stress, DNA damage and mitochondrial dysfunction that occur with aging (Palla et al., 2021; Sousa-Victor et al., 2022; Liu et al., 2022). The aging niche itself acts as an external force driving the decline in stem cell function. Specifically, the inflammatory environment created by senescence-associated secretory phenotype-derived cytokines negatively impacts the maintenance of stem cell pool (Tumpel and Rudolph, 2019; He et al., 2021).

Satellite cells indirectly regulate the structure of skeletal muscles, whereas changes in myofibers due to aging and disease directly impact muscle structure. In muscle diseases characterized by chronic inflammation, such as inclusion body myositis (IBM), functional denervation vulnerability has been observed in IBM-specific fibers (Wischnewski et al., 2024). Additionally, older individuals exhibit irregular shapes of types I and II muscle fibers compared to their younger counterparts (Soendenbroe et al., 2024).

#### 3.2.4 Mitochondria homeostasis supports muscle function

Mitochondria are highly dynamic, energy-rich organelles that meet the energy demands of muscle cells through continuous fusion and fission processes. Disruption of normal mitochondrial dynamics, such as through the knockout of key factors in mice, has been shown to result in muscle loss (Tezze et al., 2017; Favaro et al., 2019). The generation of new mitochondria, known as mitochondrial biogenesis, is largely regulated by PPAR-gamma coactivator-1 alpha (PGC-1α) (Popov, 2020). Dysregulation in the quantity and quality of mitochondria is closely associated with impaired muscle function. Skeletal muscles in individuals with sarcopenia showed remarkable signs of mitochondrial bioenergetic dysfunction, characterized by downregulation of PGC-1α/ERRα signaling, oxidative phosphorylation, and mitochondrial proteostasis genes. These alterations indicate compromised mitochondrial quality with aging (Migliavacca et al., 2019). Disuse also destroys mitochondrial homeostasis (Ji and Yeo, 2019). Mitochondrial aberrations, including mutations in mitochondrial DNA, impaired mitochondrial Ca2+ homeostasis, and disorder of mitochondrial metabolic enzymes, are key contributors to the altered mitochondrial properties observed in aging and various pathological conditions (Romanello and Sandri, 2015; Chen et al., 2023; Marzetti et al., 2024).

#### 3.2.5 NMJ degeneration and muscle strength decline

The NMJ, which connects motor nerves to muscles, mediates the force generation of skeletal muscles. Electric impulses from the presynaptic motoneuron trigger the release of acetylcholine, which then binds to its receptors on the postsynaptic membrane of the muscle fiber. This phenomenon leads to the activation of the muscle action potential and, ultimately, muscle contraction (Lepore et al., 2019). Impairment of the NMJ during aging or muscle atrophy can contribute to the decline in muscle strength (Rudolf et al., 2014). Impaired NMJ morphology can be observed as thinning of axons, synaptic detachment, fragmentation of the postsynaptic apparatus and so on (Moreira-Pais et al., 2022).

## 4 Clinical assessment methodology of muscle strength

Efficient assessment of muscle strength is essential for tracking muscle quality and diagnosing related diseases (Cruz-Jentoft et al., 2019). Muscle strength serves as a predictor of falls, mortality, and a primary parameter in the diagnosis of sarcopenia. Therefore, standardizing and unifying measurement parameters would not only improve the sharing of clinical datasets and advance research on muscle aging and disease-related functional decline globally, but also improve the convenience of population health statistics and healthcare burden forecasting.

### 4.1 Manual testing and its limitations

Clinically, manual muscle testing with certain grading systems is inexpensive, convenient, and extensively used in treatment, trials, and research. This test is used to evaluate the capability of the nervous system to adapt the muscle to meet the changing pressure of the examiners (Cuthbert and Goodheart, 2007). In the specific grading system, the subjects are asked to do the assigned movements and the score according to the evaluation their muscle performance can be a reference to the muscle situation (Bohannon, 2019). For professional clinical evaluators, manual testing can help comprehend the basic status of muscle strength and the general effect of treatments. Experienced clinical examiners and precise testing protocols are both necessary for effective manual muscle testing.

The key limitations of manual muscle testing include its reliance on experienced clinical evaluators, the subjectivity of the methodology, and the variability of the grading system. Different experts and councils globally recommend various scaling systems for muscle strength assessment. Typically, muscle strength in manual muscle testing usually is graded on a scale ranging from “no evident contraction” to “normal or full function.” However, the specific grading criteria and judgment standards can vary across systems (Bittmann et al., 2020). This subjectivity underscores the need for professional training of examiners and constant healthcare management for patients throughout the diagnosis and recovery process.

### 4.2 Dynamometer in muscle strength measurements

Measurement tools with objectivity and reproducibility are needed by scientists and clinicians to realize precise diagnosis and effective evaluation of treatment effect. The dynamometer is designed to address this demand (Table 1). Different types of exercise are involved in muscle action. Isometric exercise involves activating muscles without movement. Isotonic exercise activates muscles with a constant amount of weight. Isokinetic exercise allows muscle to exert maximum strength within the range of all joint movements at a constant speed (Kim et al., 2015; Malas et al., 2013). These exercises are applied in rehabilitation programs, clinical treatment of musculoskeletal diseases, and sports. The effects of different types of exercises on the build of muscles are regarded differently and investigated (Lee et al., 2018). Evaluation of muscle power through isometric, isotonic, or isokinetic strengthening using dynamometry provides detailed insights into muscle function and health. Isokinetic dynamometry is considered as the “gold standard” for muscle strength assessment due to its high validity, reliability, and responsiveness (Kristensen et al., 2017). Isokinetic strength measurement requires the muscle to develop dynamic force, which is regarded as more reflective to daily life activities compared with isometric measurements (Kamo et al., 2019). Take the measurement of leg muscle strength as an example. Briefly, the subjects were required to do the kicking and pulling motion in the test with the individual parameters acquired before the test. The data including peak torque, average torque and average power were collected for evaluation of muscle performance (Dean et al., 2023). The sophisticated design and organization of fixed isokinetic dynamometers make them suitable for use in healthy populations and patients with neuromuscular or other disorders (Prufer et al., 2024; Schindler et al., 2023; van der Woude et al., 2022).

**TABLE 1:** Dynamometer in muscle strength measurements.

Measurement methods	Advantages	Disadvantages
Manual testing	Inexpensive, convenient	Experienced evaluator required, subjectivity
Dynamometer	Objectivity, reproducibility	High cost

However, the implementation of isokinetic dynamometry for muscle strength assessment is restricted by several factors, including the high cost of the equipment, the need for specialized training for users, and the requirement for child-specific mechanical components when assessing pediatric populations (Mendez-Rebolledo et al., 2022).

Compared with isokinetic dynamometry, isometric dynamometry is an alternative that is relatively easy to handle, portable, and inexpensive. These characteristics of the isometric dynamometry device promoted its application in various scenarios, including scientific research and clinical settings (De Blaiser et al., 2018). With the guidance of testers, movements of the interested muscles were performed for a certain period with a few times of attempts. The maximum strength was collected to evaluate the muscle performance (Fortes et al., 2023). In a clinical study comparing hand-held isometric dynamometry and the fixed, laboratory-based dynamometry with the function of isokinetic assessment, hand-held dynamometry showed excellent reliability and validity for most assessments of lower limb strength and power in the tested healthy population (Mentiplay et al., 2015). Hand-grip dynamometry can provide information regarding isometric grip strength, which is difficult to assess for hand-held dynamometry (Mendez-Rebolledo et al., 2022).

## 5 Approaches for improving muscle strength loss

### 5.1 Resistance training and improvement of muscle quality

With muscle contracting against external resistance, resistance training is an effective exercise for building muscle mass in young and older populations (Liao et al., 2019). A systematic review summarized that, although exercise programs may not significantly increase muscle mass in sarcopenic older adults, they do have a positive effect on muscle strength and physical performance (Bao et al., 2020). Progressive resistance training is directly correlated with increased muscle strength (O'Bryan et al., 2022). Studies have indicated that resistance training can increase the satellite cell content in muscles, particularly in type II fibers (Heidari et al., 2023). One study involving 1,600 healthy, community-dwelling older individuals explored the impact of exercise habits during adolescence and later in life on the risk of sarcopenia with aging. Subjects were divided into four groups according to their exercise habits in adolescence and older age, including people without exercise habits in neither, anyone, nor both periods. The assessment of their muscle quality showed that older men and women with exercise habits in adolescence and older age were at a lower risk of sarcopenia and low muscle performance (Tabata et al., 2023).

### 5.2 Aerobic exercise and muscle strength

Unlike resistance exercise, aerobic exercise, such as jogging and walking, involves physical activities based on aerobic metabolism (Irawati et al., 2024). Although aerobic exercise has been previously known to positively affect the overall physical health, it may not influence muscle size. However, emerging pieces of evidence demonstrate that aerobic exercise with appropriate intensity, duration, and frequency can lead to muscle hypertrophy and/or enhanced muscle function (Konopka and Harber, 2014). A study observing the influence of aerobic exercise on older adults utilizing dual-energy X-ray and isokinetic dynamometer reported that an aerobic training lasting for 24 weeks led to increased myofibrillar protein synthesis and muscle strength in the exercise group compared with the non-exercise group; however, lean mass was minimally changed by the exercise intervention (Brightwell et al., 2019). Another human study involving subjects ranging from 20 to over 80 years-old found that compared with sedentary adults, the highly aerobic active men and women showed higher grip strength relative to body weight and greater leg lean mass (Crane et al., 2013).

Compared with exercise training interventions for healthy young and older populations to improve their muscle health, exercise strategies for patients with neuromuscular and musculoskeletal disorders require additional healthcare support. Individuals with muscle diseases are likely trapped in a vicious circle due to physical inactivity. Exercise training is also commonly prescribed to decelerate muscle mass and strength loss and improve muscle function. Therefore, exercise programs should be carefully designed individually by experienced clinicians and practitioners (Bao et al., 2020; Voet, 2019; Cavaggioni et al., 2024; Concha-Cisternas et al., 2023).

### 5.3 Dietary adjustment and muscle strength

The blunted response to dietary stimulation and the reduced intake of dietary nutrition contribute to impaired muscle protein synthesis and muscle function. Thus, intervention with dietary adjustment and nutrition supplementation may improve muscle homeostasis and physical performance of the elderly and other individuals with degenerated muscles. Dietary intervention is easier to implement compared with persistent exercise training. Research on elderly men and women showed notably increased muscle protein turnover after a rise in protein content of the diet (Pannemans et al., 1995). Although no substantial change in protein turnover was observed after a period of a low-protein diet, inadequate protein intake resulted in impaired nitrogen balance and diminished lean mass and muscle function (Castaneda et al., 1995a; Castaneda et al., 1995b). In addition to protein, Vitamin D has also been proposed to be beneficial to muscle strength. Vitamin D insufficiency is associated with decreased muscle function and increased risk of disability, whereas its supplementation improves muscle strength and gait performance, especially in elderly individuals (Halfon et al., 2015). Protein intake is one of the key processes of muscle mass maintenance and increase in dietary intervention, which decreases during aging and contributes to the deterioration of muscle quality in sedentary and older adults (Dicks et al., 2020). Sufficient food supply is indispensable for healthy muscle strength. However, energy and nutritional balance are the ultimate goal of dietary intervention, rather than solely supplementing any specific nutritional component. Long-term negative energy balance decreased muscle mass and performance due to several pathways, including suppressed metabolic rate, decreased protein turnover, and so on (Carbone et al., 2012). In addition to protein, a balanced diet with a healthy food intake pattern, as well as the intake of other nutrients, including antioxidant and anti-inflammatory nutrients, are also crucial for muscle strength and physical performance (Robinson et al., 2019). Given that dietary nutrition and exercise can interact synergistically to promote muscle health, a combination of improved nutrition and exercise training presents a promising approach to enhance muscle strength (Kim et al., 2020). From the evidence up to now, both appropriate exercise training and dietary intervention are beneficial to muscle quality. When it comes to the influence of combined dietary intervention and exercise on muscle mass and strength, however, whether there is a synergic effect on the approach is still remained unclear (Choi et al., 2021; Voulgaridou et al., 2023). More clinical observation and evaluations are needed to answer this question. The interventions aimed at improving muscle strength are summarized in Table 2.

**TABLE 2:** Strategies for muscle improvement.

Exercise	Resistance exercise
	Aerobic exercise
Dietary intervention	Increase protein content
	Vitamin D supplement
	Energy and nutritional balance

## 6 Practical applications

The progressively developed technologies of data collection and analysis in clinical research have largely expanded the knowledge regarding the factors associated with muscle strength loss. In contrast, scientific research of skeletal muscle with the assistance of single-cell multi-omics has strengthened the comprehensive understanding of the growth, aging, and regeneration of skeletal muscle. The combination and mutual promotion of achievements from scientific investigation and clinical intervention are anticipated in the future.

For muscle strength assessment, tools including manual testing, isometric dynamometry, and isokinetic dynamometry demonstrate advantages and disadvantages. For most parts of the world, especially underdeveloped areas, the popularization of fixed isokinetic dynamometry can be difficult in the short term. Therefore, design optimization and utilization popularization of the portable isometric dynamometry are urgently needed for health monitoring due to muscle strength, which is an indicator of physical health.

The positive effects of exercise and nutrition supplementation on muscle strength have been widely accepted. However, additional clinical observation and statistical evidence with standardized intervention are still required in the future.
